# MLNGCF: circRNA–disease associations prediction with multilayer attention neural graph-based collaborative filtering

**DOI:** 10.1093/bioinformatics/btad499

**Published:** 2023-08-10

**Authors:** Qunzhuo Wu, Zhaohong Deng, Wei Zhang, Xiaoyong Pan, Kup-Sze Choi, Yun Zuo, Hong-Bin Shen, Dong-Jun Yu

**Affiliations:** School of Artificial Intelligence and Computer Science, Jiangnan University, Wuxi, China; School of Artificial Intelligence and Computer Science, Jiangnan University, Wuxi, China; School of Artificial Intelligence and Computer Science, Jiangnan University, Wuxi, China; Institute of Image Processing and Pattern Recognition, Shanghai Jiaotong University, Shanghai, China; The Centre for Smart Health, The Hong Kong Polytechnic University, Hong Kong; School of Artificial Intelligence and Computer Science, Jiangnan University, Wuxi, China; Institute of Image Processing and Pattern Recognition, Shanghai Jiaotong University, Shanghai, China; School of Computer Science and Engineering, Nanjing University of Science and Technology, Nanjing, China

## Abstract

**Motivation:**

CircRNAs play a critical regulatory role in physiological processes, and the abnormal expression of circRNAs can mediate the processes of diseases. Therefore, exploring circRNAs–disease associations is gradually becoming an important area of research. Due to the high cost of validating circRNA–disease associations using traditional wet-lab experiments, novel computational methods based on machine learning are gaining more and more attention in this field. However, current computational methods suffer to insufficient consideration of latent features in circRNA–disease interactions.

**Results:**

In this study, a multilayer attention neural graph-based collaborative filtering (MLNGCF) is proposed. MLNGCF first enhances multiple biological information with autoencoder as the initial features of circRNAs and diseases. Then, by constructing a central network of different diseases and circRNAs, a multilayer cooperative attention-based message propagation is performed on the central network to obtain the high-order features of circRNAs and diseases. A neural network-based collaborative filtering is constructed to predict the unknown circRNA–disease associations and update the model parameters. Experiments on the benchmark datasets demonstrate that MLNGCF outperforms state-of-the-art methods, and the prediction results are supported by the literature in the case studies.

**Availability and implementation:**

The source codes and benchmark datasets of MLNGCF are available at https://github.com/ABard0/MLNGCF.

## 1 Introduction

With the development of sequencing technology and bioinformatics, circRNA have been discovered abundant in eukaryotic cells (Holdt *et al.* 2018, Wesselhoeft *et al.* 2018) with increasing diversity. For example, circRNA_100395 acts as a microRNA sponge in breast cancer pathogenesis to suppress overexpression of the gene MAPK6 (Yu *et al.* 2020), inhibiting the proliferation and expansion of breast cancer cells. There is increasing evidence showing that the circRNAs are associated with many diseases, and thus it was extensively recognized as a biomarker for predicting diseases (Vuolteenaho *et al.* 2005, Li *et al.* 2015) with therapeutic effects (Lei *et al.* 2019, Verduci *et al.* 2019, Liu *et al.* 2022).

In recent years, a number of related databases are established for circRNAs, diseases, and circRNA–disease associations. The databases about circRNA are CircNet (Chen *et al.* 2022), deepBase (Yang *et al.* 2010), circBase (Glažar *et al.* 2014), etc. The databases about diseases are Mesh (Mottaz *et al.* 2008), and MalaCards (Rappaport *et al.* 2013), etc. The databases about the circRNA–disease associations between circRNA and diseases are circR2Disease (Fan *et al.* 2022), circ2Disease (Yao *et al.* 2018) and circRNADisease (Zhao *et al.* 2018).

Although the above high-quality databases of circRNA–disease associations have been developed, a large number of circRNA–disease associations still unknown. With the rapid development of machine learning methods (Chen *et al.* 2022), the usage of these techniques to predict unknown circRNA–disease associations has become a popular topic. These methods can be broadly divided into two groups. The first group of methods pays more attention to enriching the input embeddings. For example, Wang *et al.* proposed the IMS-CDA (Wang *et al.* 2020), which combined the disease semantic similarity, disease Jaccard similarity, Gaussian interaction profile kernel similarity and circRNA similarity information to extract the hidden features using Stacked Auto-Encoder (SAE). Wei *et al.* proposed iCircDA-MF (Wei and Liu, 2020) to introduce gene information into the limited size of the training data and construct the circRNA–gene–disease relation network to expand the data sources, this model used matrix factorization and completion techniques to reduce the feature noise. Chen *et al.* proposed RGCNCDA (Chen *et al.* 2022), in which a circRNA–miRNA–disease global heterogeneous network is first constructed by integrating three biological entity networks based on relational convolutional networks (R-GCNs).

The second group of methods pays more attention to feature extraction. For example, Wang *et al.* proposed the GCNCDA (Wang *et al.* 2020) by introducing FastGCN for fast extraction of high-order features and using Forest by Penalizing Attributes (Forest PA) classifier for making a prediction. Zheng *et al.* proposed the ICDA-CGR (Zheng *et al.* 2020), which introduced circRNA sequence information and quantified the nonlinear relationship in circRNA sequences by Chaos Game Representation (CGR) based on the sequence position information. The SIMCCDA (Li *et al.* 2020) propose matrix completion to predict the associations between circRNAs and diseases by Speedup Inductive Matrix Completion (SIMC). Niu *et al.* proposed the GMNN2CD (Niu *et al.* 2022), which employed a graph Markov convolutional neural network to score the potential circRNA–disease associations by integrating a graph autoencoder and variational inference. Bian *et al.* proposed the GATCDA (Bian *et al.* 2021) model, which utilized a graph attention network (GAT) to predict circRNA–disease associations with disease symptom similarity, network similarity, and information entropy similarity for both circRNAs and diseases. Zhao *et al.* proposed the IBNPKATZ (Zhao *et al.* 2019), which integrated the bipartite network projection algorithm and KATZ measure to achieves reliable prediction. Ge *et al.* proposed LLCDC (Ge *et al.* 2020), which reconstructed similarity networks using Locality-Constrained Linear Coding (LLC) on the known association matrix. Furthermore, some state-of-the-art computational models and publicly accessible databases are summarized by Wang *et al.* (2021).

However, the above mentioned methods still have some limitations. The first group of methods focuses too much on the construction of circRNAs and disease similarity and ignores the connection and collaborative signals hidden in the circRNA–disease networks. The second group of methods is difficult to distill the desired collaborative signals in the circRNA–disease network (Wang *et al.* 2019).

To solve the challenges discussed above, we propose a graph collaborative filtering method MLNGCF based on multilayer attention and collaborative filtering. MLNGCF refines the similarity information as initial features of nodes on the central network and proposes a new message propagation network with a multilayer cooperative attention mechanism. In this network, the first-order connectivity model is expanded by stacking more message propagation layers to explore high-order connectivity messages. In addition, a multilayer cooperative attention mechanism is introduced to further weigh the messages propagated at different layers. Finally, a collaborative filtering model is used for the prediction of circRNA–disease associations. Five-fold cross-validation results on the benchmark datasets show that MLNGCF performs better than existing methods, and the prediction results from case studies are also supported by the literature.

## 2 Materials and methods

### 2.1 The framework of the proposed model MLNGCF

**Figure 1.**
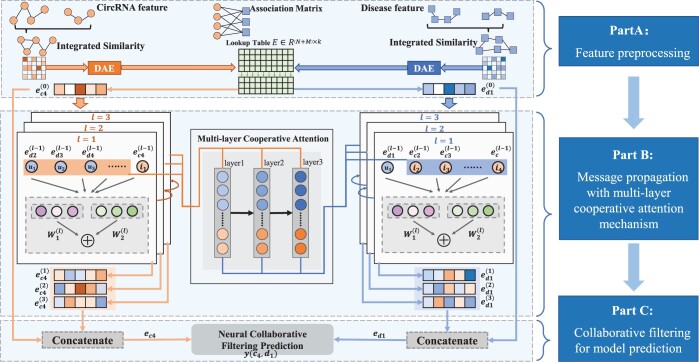
The framework of the proposed MLNGCF. It can be divided into three parts. In part A, uniform representations of circRNAs and disease are obtained after feature preprocessing and a circRNA–disease lookup table is constructed. In part B, a message propagation with cooperative attention mechanism is proposed to optimize the representations of circRNAs and disease. In part C, a neural collaborative filtering predictor is constructed for making prediction. The part B and part C are displayed in the form of node to node for easy understanding.

The framework of the proposed MLNGCF is illustrated in Fig. 1. First, the semantic similarity of diseases, the functional similarity of circRNAs, the GIP kernel similarity of circRNAs, and the GIP kernel similarity of diseases are calculated and refined as the initial embeddings of circRNAs and diseases. Second, the initial embeddings are propagated with multilayer cooperative attention on the circRNA–disease adjacency matrix to generate higher-order embeddings of circRNAs and diseases. Finally, circRNA–disease associations are predicted by a collaborative filtering model to approximate the initial circRNA–disease adjacency matrix as closely as possible.

### 2.2 Benchmark datasets

In this article, we collect three public databases (circR2Disease, circ2Disease, and circRNADisease) as a unified dataset of circRNA–disease associations to measure the model performance. CircR2Disease is a collection of numerous experimentally validated circRNA–disease associations, which contains a total of 739 circRNA–disease associations between 661 circRNAs and 100 diseases. After removing redundant data, we obtain 650 circRNA–disease pairs between 585 circRNAs and 88 diseases. Similarly, the circ2Disease database contains a total of 270 circRNA–disease associations between 249 circRNAs and 60 diseases. circRNADisease database contains a total of 350 circRNA–disease associations between 330 circRNAs and 48 diseases. In this article, the strategy of Wang *et al.* (2020) is used to construct the negative samples, we construct the final dataset with the same number of positive samples and negative samples.

We also use the PubMed (Canese and Weis 2013) medical literature database and Mesh database for diseases. The Mesh database is a database of disease relationships deposited in the form of directed acyclic graphs. The PubMed database comprises more than 35 million citations for biomedical literature.

Based on the CircR2Disease, circ2Disease and circRNADisease databases, we construct the circRNA–diseases adjacency matrix, which has the size of N×M, corresponding to N circRNAs and M diseases, respectively. If a circRNA is related to a disease, the value in the corresponding adjacency matrix is 1, otherwise 0.

### 2.3 Similarity construction

After obtaining the adjacency matrix, four similarity matrices are calculated, they are disease semantic similarity matrix; disease GIP kernel similarity matrix; circRNA functional similarity matrix; and circRNA GIP kernel similarity matrix. The details for the construction of the above similarity matrices are described in Supplementary Parts SA–SD.

In order to facilitate the embedding construction, the features of circRNAs and diseases are fused to form the initial descriptor. The descriptor not only reveals the associations between circRNAs and diseases but also represents the hidden connections between circRNAs and diseases.

Here, we use a new disease descriptor defined in Niu *et al.* (2022) and Wang *et al.* (2020). If there is a semantic similarity association between two diseases d(i) and d(j), then the disease similarity descriptor $\mathrm{DSim}\left(d(i),d(j)\right)$ is defined as the semantic similarity between the two diseases; otherwise, it is defined as the GIP kernel similarity of diseases. The detailed calculation is as follows: where $\mathrm{SD}\left(d\left(i\right),d\left(j\right)\right)$ and $\mathrm{DGS}\left(d\left(i\right),d\left(j\right)\right)$ represent semantic similarity and GIP kernel similarity of disease i and disease j.


(1) DSimci,cj=SDdi,dj      if di and dj has semantic similarityDGSdi,dj otherwise

Similarly, the functional similarity of circRNAs and GIP kernel similarity constructed for circRNAs are used to form new circRNA similarity descriptor $\mathrm{CSim}\left(c(i),c(j)\right)$: where $\mathrm{FC}\left(c\left(i\right),c\left(j\right)\right)\;$and $\mathrm{CGS}\left(c\left(i\right),c\left(j\right)\right)$ represent functional similarity and GIP kernel similarity of circRNA i and circRNA j.


(2) CSimci,cj=FCci,cj if ci and cj has functional similarityCGSci,cj otherwise

### 2.4 Feature preprocessing

Deep autoencoder is an unsupervised neural network that projects data from a high dimension to a low dimension (Chicco *et al.* 2014, Tan *et al.* 2016). In this study, a deep autoencoder is proposed to generate a uniform representation of circRNAs and diseases.

For the similarity construction, the descriptor of circRNAs CSim and the descriptor of related diseases DSim are obtained. Take similarity features of diseases as an example, the encoding operation of the autoencoder can be expressed as: where where w and b are the weight and bias, respectively.


(3) Ds=Lw⋅DSim+b

(4) Lx=11+ exp ⁡(-x)

After obtaining the embedding Ds, the decoding operation of the autoencoder is constructed using a similar approach: where DSim' represents the descriptor of diseases after autoencoder refactoring. w' and b' denotes the weight and bias of the decoding operator, respectively.


(5) DSim′=L=w'⋅Ds+b'

In order to obtain the high-level features of diseases, this operation will stop until $\mathrm{DSi}m^{'}$ is approximately equal to DSim. Then, the learned embedding $Ds\in R^{M\times k}$ is used as the new disease similarity feature matrix, where k denotes the feature dimension and it is set as 128 in this article. Similarly, the embedding of circRNAs can also be obtained in the same way.

### 2.5 Message propagation with multilayer cooperative attention mechanism

In order to capture the hidden collaborative signals in a graph structure, we established a GNN-based message propagation mechanism based on the model proposed by Wang *et al.* (Wang *et al.* 2019, Sun *et al.* 2022). To further enhance the discriminability of the learned embeddings, we propose a single-layer message propagation mechanism on the central network. In addition, a multilayer cooperative attention mechanism is added to optimize the embedding process in the multilayer message propagation to coordinate the weights among different layers.

#### 2.5.1 Embedding lookup table construction

After the autoencoder reconstruction, the initial feature matrix of circRNAs Cs and the initial feature matrix of diseases Ds are obtained. We denote $e_{c}\in R^{k}$, $e_{d}\in R^{k}$ as the column of circRNA feature matrix Cs and disease feature matrix Ds, respectively. Then, an embedding lookup table can be constructed as follows: where N is the number of different circRNAs, M is the number of different diseases.


(6) E=ec1,…,ecN, ed1,…,edM∈RN+M×k

Then, we set this embedding lookup table as the input of the training model in message propagation. Different from traditional matrix factorization, the embeddings of circRNA and disease are refined by propagating over the circRNA–disease association network. Since the association network can fully incorporate collaborative signals when compared with matrix factorization-based methods, the more discriminable embeddings of circRNAs and diseases can be obtained in the proposed method.

#### 2.5.2 Single-layer message propagation

In traditional recommendation systems, the user who buys an item can be considered as a feature of that item, this feature can be used to measure the similarity between two items (Ko *et al.* 2022), since the interaction between the item and the user directly indicates the user’s preferences. Similarly, this approach is also applicable to measure the associations of circRNAs and diseases. Thus, a mechanism is established for message propagation between circRNAs and diseases by dividing it into two main processes: message construction and message aggregation.

##### (a): message construction

Given a circRNA–disease pair (c,d) in circRNA–disease association network, a message propagation mechanism from c to d can be established as follows: where $m_{d\leftarrow c}$ is the message (embeddings to be propagated) passed from c to d. $f\left(\cdot \right)$ denotes the encoding function of the message, which takes embeddings $e_{c}$ and $e_{d}$ as input. $p_{dc}$ is the coefficient factor to control the decay rate in this process. Equation (7) can be further expressed as follows: where $1/\sqrt{\left|N_{d}\right|+\left|N_{c}\right|}$ (equals to $p_{dc}$) is the graph Laplacian norm with $N_{c}$ and $N_{d}$, denoting the first-hop neighbors of circRNA c and disease d. It can be interpreted as a discount factor because the messages being propagated should decay with the path length. $W_{1}\in R^{k^{'}\times k}$ and $W_{2}\in R^{k^{'}\times k}$ are trainable weight matrices to extract useful messages in the propagation process. $k^{'}$is the size of messages after transformation.


(7) md←c=fec,ed,pdc

(8) md←c=1Nd+NcW1ec+W2ec⊙ed

In Equation (9), not only the contribution of node $e_{c}$ can be considered but also the correlation between $e_{c}$ and $e_{d}$ can be enhanced through $e_{c}⊙e_{d}$, which creates a dependence between $e_{c}$ and $e_{d}$ in message propagation.

##### (b): message aggregation

In message construction, the propagated messages on the circRNA–disease path are constructed. Take the central network of disease as an example, the messages passed from the neighboring nodes of a disease are aggregated and refined to form the embedding representations. The aggregation function is defined as: where $e_{d}^{\left(1\right)}$ denotes the embeddings of disease d obtained after the first message propagation, $m_{d\leftarrow d}=W_{1}e_{d}$, $m_{d\leftarrow c}=W_{1}e_{c}.\;W_{1}\;$is the same as $W_{1}$ in Equation (8). Similarly, the aggregation function $e_{c}^{\left(1\right)}$ for the circRNA can be built by the disease nodes in the central network of circRNA c. Equation (9) not only considers the messages propagated from neighboring nodes $N_{d}$ to disease d but also the connection of disease d itself.


(9) ed1=LeakyReLUmd←d+∑c∈Ndmd←c

#### 2.5.3 Multilayer message propagation

**Figure 2.**
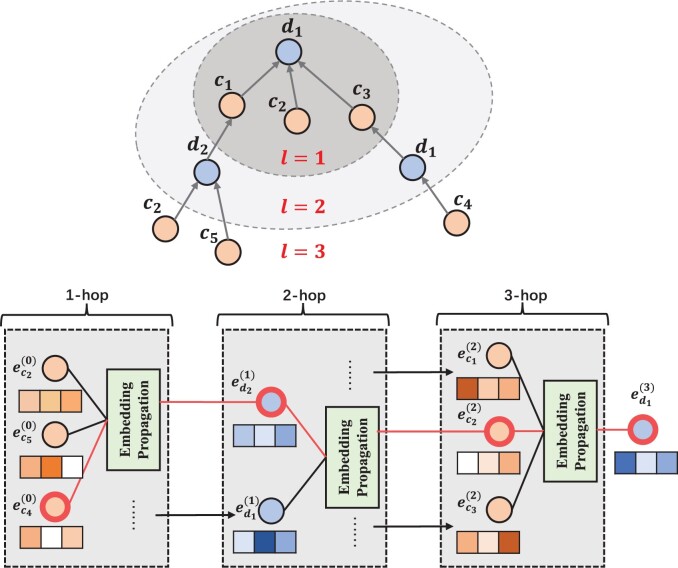
Detailed flowchart of the three-layer message propagation used in MLNGCF with the central network of disease $d_{1}$ as an example. The path from $e_{c_{4}}^{\left(0\right)}$ to $e_{d_{2}}^{\left(1\right)}$ to $e_{c_{2}}^{\left(2\right)}$ to $e_{d_{1}}^{\left(3\right)}$ is an example path.

Based on the above single-layer (single-order) message propagation, more message propagation layers can be stacked to explore higher-order connectivity information, and this higher-order connectivity signal can be used to measure the correlation between circRNAs and diseases. In this article, as shown in Fig. 2, by stacking l message propagation layers, the central disease or circRNA is able to receive messages from l-layer neighboring nodes. When propagating to layer l, the embedding of disease d can be formulated as: where the messages propagated are defined as follows: where $W_{1}^{\left(l\right)},W_{2}^{\left(l\right)}\in R^{k_{l}\times k_{l-1}}$ are trainable transformation matrices, $k_{l}$is the size of messages after transformation. $e_{c}^{\left(l-1\right)}$ represents the embeddings after (l-1) times message propagation.


(10) edl=LeakyReLUmd←dl+∑c∈Ndmd←cl

(11) md←cl=pdcW1lecl-1+W2lecl-1⊙edl-1md←dl=W1ledl-1 

Analogously, the embeddings of related diseases can also be obtained by applying the same mechanism of message propagation. The detailed process is shown in Fig. 2. In addition, during message propagation, different neighbors may contribute differently to the central node. In this article, a multilayer collaborative attention mechanism is introduced to capture the contribution values of different neighbors before message aggregation and update the representations of the central nodes.

#### 2.5.4 Multilayer cooperative attention mechanism on message propagation

**Figure 3.**
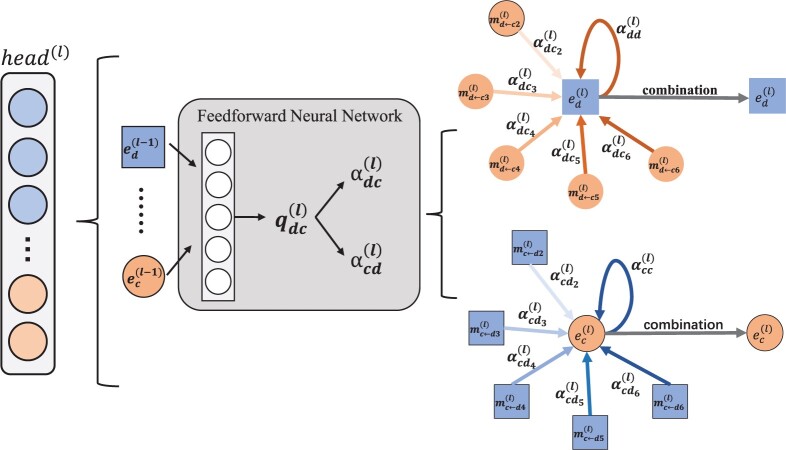
The implementation process of the l-th layer attention head in the multilayer cooperative attention mechanism.

For a central node, the embeddings of neighbors are first calculated, and then the embeddings of a central node are reconstructed after message propagation. It is worth noting that during the process, the message weights of different nodes in the same layer are identical, controlled by $p_{dc}$. which cannot capture the contributions of different nodes in the same layer. Therefore, the GAT model (Veličković *et al.* 2017) is used to learn the weights of different nodes in the same layer. However, GAT ignores the dependency between different attention heads. To this end, we propose multilayer cooperative attention, which allows different attention heads to be distributed in different message layers to establish their relationship. The detailed process is shown in Fig. 3.

First, the attention score of circRNA and disease is calculated based on the message propagation: where, f() represents the single-layer feedforward neural network, and W is the weight matrix of the network. The network transforms the input into the embeddings of circRNAs and diseases. $e_{d}^{\left(l-1\right)}$ and $e_{c}^{\left(l-1\right)}$ denotes the embedding of disease and circRNA in the l-th layer, respectively.


(12) qdcld,c=fWedl-1,Wecl-1

Then, the attention scores are normalized by the following process: where $N_{d}$ represents the neighbors of the central node of disease d in the *l*-th layer. $\alpha_{dc}^{\left(l\right)}$ is the contribution value of circRNA c to disease d during message propagation.


(13) αdcl=exp⁡LeakyReLUqdcl∑t∈Ndexp⁡LeakyReLUqdtl

The linear combination of the central network is used to update the embedding of disease d: where LeakyReLU represents activation function, $\alpha_{dc}^{\left(l\right)}$ denotes the attention scores of neighbors in central networks of disease d.


(14) edl=LeakyReLUmd←dl+∑c∈Ndαdclmd←cl

According to Equation (13), the attention mechanism on the central network can be implemented in the process of message propagation at each layer. Therefore, each propagation layer corresponds to an attention head, and as the number of layers increases, richer representational information can be learned. This mechanism of message propagation between different layers builds the dependency in different attention heads.

After obtaining the weighted embeddings of circRNAs and diseases, the hierarchical propagation rule is proposed: where $E^{\left(l\right)}\in R^{(N+M)\times k_{l}}$ is the embeddings of circRNAs and diseases after *l*-th message propagation. The initial value of embedding representation $E^{\left(0\right)}$ before message propagation is E, where $e_{d}^{\left(0\right)}=e_{d}\;$and $e_{c}^{\left(0\right)}=e_{c}$. I is the identity matrix, and L is the Laplacian matrix of circRNA–disease association matrix: where $R\in R^{N\times M}$ denotes the circRNA–disease association matrix, 0 denotes the zero matrix, $A\in R^{\left(N+M)\times (N+M\right)}$ denotes the adjacency matrix, and D denotes the diagonal matrix and its diagonal element $D_{tt}=\left|N_{t}\right|$. $\theta^{\left(l\right)}\in R^{\left(N+M)(N+M\right)}$ is the weight matrix of *l-*th layer cooperative attention and there is $\theta^{\left(l\right)}=\left[\alpha_{11}^{\left(l\right)},\alpha_{12}^{\left(l\right)},\ldots ,\alpha_{\left(N+M)(N+M\right)}^{\left(l\right)}\right]$. By implementing propagation rules, the embeddings of circRNAs and diseases can be updated simultaneously.


(15) El=LeakyReLUEl-1W1l+θl-1LEl-1W1l+θl-1LEl-1⊙El-1W2l

(16) L=D-12AD12 and A=0RRT0

### 2.6 Collaborative filtering for model prediction

Based on the above processes, the multiple representations $\left\{e_{c}^{\left(1\right)}\ldots e_{c}^{\left(l\right)}\right\}$ and $\left\{e_{d}^{\left(1\right)}\ldots e_{d}^{\left(l\right)}\right\}$ for circRNAs and diseases are obtained. Since the embeddings propagated from different layers do not contain the same content and the contributions are different. Referring to Wang’s method (Wang *et al.* 2019), the embeddings of different layers are concatenated to form the final representations of circRNAs and diseases:


(17) ec*=ec1‖…‖ecled*=ed1‖…‖edl#

Thus, the final embeddings of circRNAs and diseases are obtained. In order to fully utilize the embeddings to calculate the association scores between circRNAs and diseases (He *et al.* 2017), a neural collaborative filtering is proposed to predict the associations between circRNAs and diseases.

In the collaborative filtering network, matrix factorization (MF) (Koren *et al.* 2009, Li *et al.* 2020) and multilayer perceptron (MLP) (Ramchoun *et al.* 2016) are introduced as an instance of interaction function to infer the potential associations between circRNAs and diseases.

Generalized Matrix Factorization (GMF) (Lee and Seung 2000, Shan and Banerjee 2010, Ma and Liu 2022) is widely used in collaborative filtering for recommendation. Generally, the input of the model is a one-hot encoded representation, which is fed into one fully connected layer to generate the dense vector of circRNAs or diseases. The first mapping layer of the GMF is defined as follows: where ⊙ denotes the element-wise product of vectors.


(18) Egmfec*,ed*=ec*⊙ed*

To take the nonlinear relationship between circRNAs and diseases into consideration, a standard MLP is introduced to learn the interactions of latent features and improve the nonlinear modeling capabilities. The neural collaborative filtering is defined as follows: where $W_{i},a_{i},b_{i}(i\in 1,2,\ldots L)$ denote the weight matrix, ReLU activation function, and bias of the layer i, respectively.


(19) Emlp=aLWLTaL-1…a1W1Tec*ed*+b1…+bL

**Figure 4.**
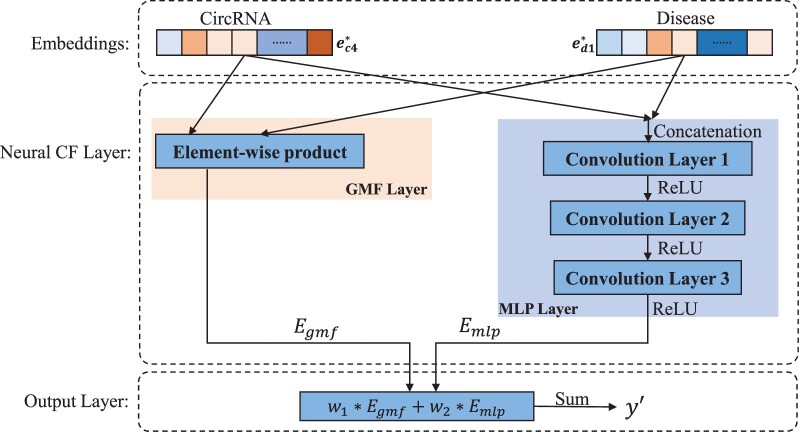
An illustration of the interaction function of collaborative filtering.

GMF applies a linear kernel to model the latent features, and MLP uses a non-linear kernel to learn the interaction function from data. In order to make the prediction model with both linear and nonlinear learning capabilities, GMF with a one-layer MLP is used as follows: where $E_{\mathrm{gmf}}$ and $E_{\mathrm{mlp}}$ denote the outputs of $e_{c}^{\left(*\right)}$ and $e_{d}^{\left(*\right)}$ after matrix decomposition and MLP operation, respectively. h denotes the connection weights of matrix factorization and MLP. Here, the sum of vector elements instead of activation functions is used for mapping. The detailed process of collaborative filtering is shown in Fig. 4.


(20) y'=sumhTEgmfEmlp

### 2.7 Parameter optimization of MLNGCF

Based on the hypothesis that confirmed circRNA–disease pairs reflect the associations between circRNAs and diseases, MLNGCF assigns a high score to confirmed circRNA–disease pairs and a low score to unknown circRNA–disease pairs. The optimized objective function is defined as follows: where $S=\left\{(c,i,j)|(c,i)\in S^{+},(c,j)\in S^{-}\right\}$ denotes the paired training data. $S^{+}$and $S^{-}$ denote the confirmed circRNA–disease associations and unknown circRNA–disease pairs, respectively. i and j represent two disease belongs to $S^{+}$and $S^{-}$, respectively. σ denotes the activation function. Θ denotes the trainable parameters. λ is the regularization parameter. Specifically, Adam is used as the optimizer for model training.


(21) Loss=∑c,i,j∈S-ln⁡σyci'-ycj'+λΘ22

## 3 Results

### 3.1 Evaluation metrics

In this study, a 5-fold cross-validation approach is used to evaluate the performance of the prediction method MLNGCF. All known circRNA–disease associations are evenly divided into *k* subsets, where each subset takes turn to be the test set, while the other subsets are used as the training set. In this study, area under the receiver operating characteristic curve (AUC), area under the precision-recall curve (AUPR), and accuracy are used as the performance metrics. In addition, F1-score and NDCG (Wang *et al.* 2013) are used. The details of each metric are described in Supplementary Part SF.

### 3.2 Performance of MLNGCF on circR2Disease

**Figure 5.**
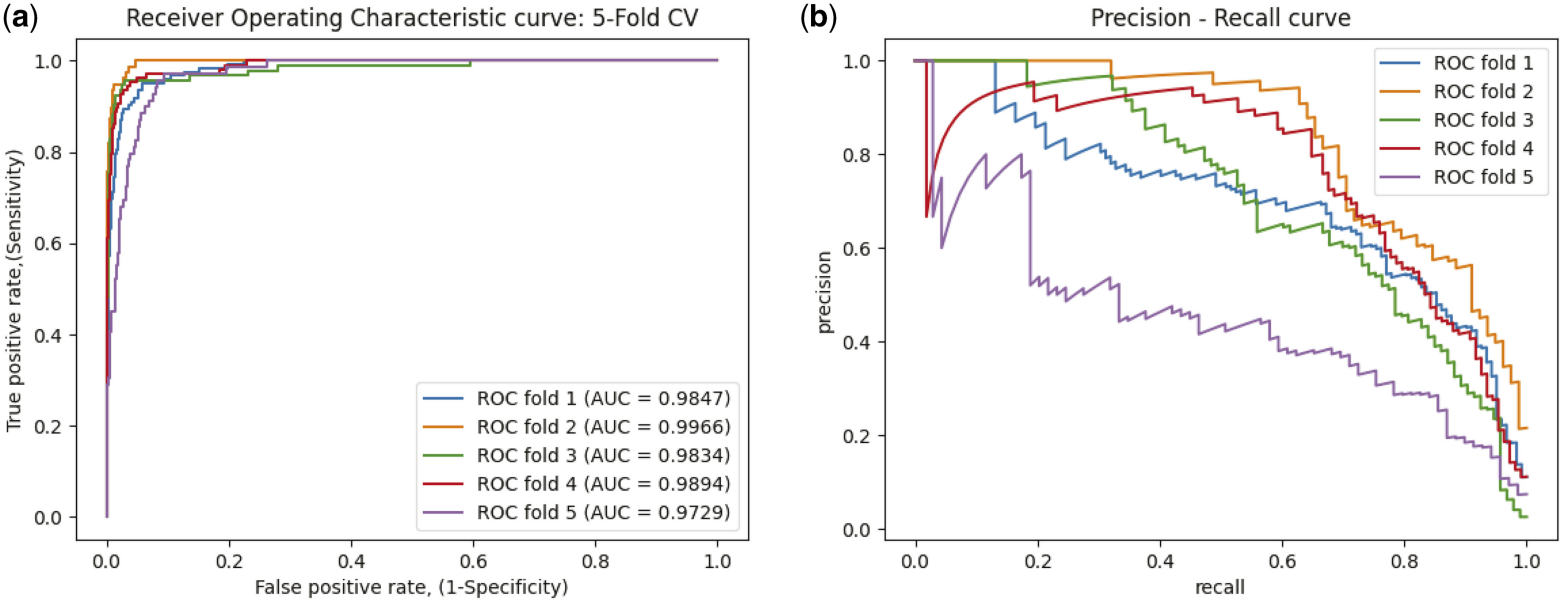
The ROC curves of 5-fold cross-validation by the proposed MDGF-MCEC on the circR2Disease database. (a) TPR–FPR curve; (b) precision–recall curve.

As 5-fold cross-validation approach is used for evaluating the performance of MLNGCF, which produces the final results in an averaged manner. The ROC curves and the precision–recall curves obtained for each fold in the experiments are shown in Fig. 5. The rest of the metrics are given in Table 1.

**Table 1.** Results of 5-fold cross-validation obtained by the proposed MLNGCF on circR2Disease.

Validation set	ACC. (%)	AUPR (%)	AUC (%)	F1 (%)	NDCG (%)
1	97.83	69.64	98.47	67.56	92.42
2	97.56	86.98	99.66	78.65	96.44
3	98.15	70.65	98.34	74.22	94.51
4	96.58	74.47	98.94	65.07	91.65
5	97.21	64.74	97.29	56.68	88.45
Average	97.46 ± 0.60	72.49 ± 8.39	97.29 ± 0.60	68.43 ± 8.48	88.69 ± 3.02

As shown in Table 1, It can be seen that the proposed method MLNGCF achieves promising results. The key metrics AUC is 98.54% and AUPR is 72.49%. However, it can also observe that the model still yields fluctuations in F1-scores and AUPR for different folds, which may be caused by the limited available data.

**Figure 6.**
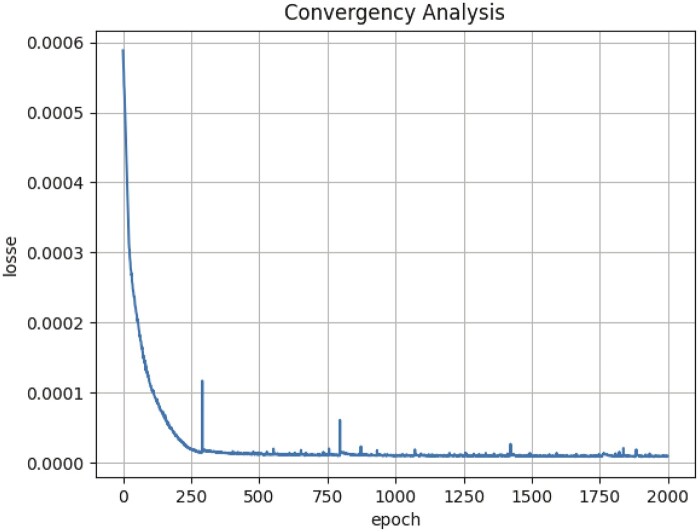
The training loss curve generated on circR2Disease databases after 2000 epochs.

Furthermore, the loss curve is drawn to verify the convergence of the proposed model under circR2Disease database in Fig. 6. It can be seen that proposed model can converge after 750 iterations.

### 3.3 Comparison with state-of-the-art methods

To demonstrate the advantages of the proposed model MLNGCF, we compare it with existing representative methods on the circR2Disease database. The compared methods are RGCNCDA (Chen *et al.* 2022), GCNCDA (Wang *et al.* 2020), GMNN2CD (Niu *et al.* 2022), iCircDA-MF (Wei and Liu 2020), IMS-CDA (Wang *et al.* 2020), and ICDA-CGR (Zheng *et al.* 2020). Since the evaluation metrics adopted by different methods are different, the main evaluation metric AUC is chosen here for comparison, and the results are given in Table 2. It should be noted that although the methods under the comparison are all evaluated on the circRNA–disease associations from the circR2Disease database, the data that they used are not completely the same. For example, iCircDA-MF uses only human data, while GCNCDA used both human data and the data from other species.

**Table 2.** Performance comparison of MLNGCF with six state-of-the-art methods in terms of AUC.

Methods	MLCNGCF (our method)	GMNN2CD	RGCNCDA	iCircDA-MF	GCNCDA	IMS-CDA	ICDA-CGR
AUC	0.973	0.963	0.949	0.918	0.910	0.881	0.853

### 3.4 Performance on circ2Disease and circRNADisease

**Figure 7.**
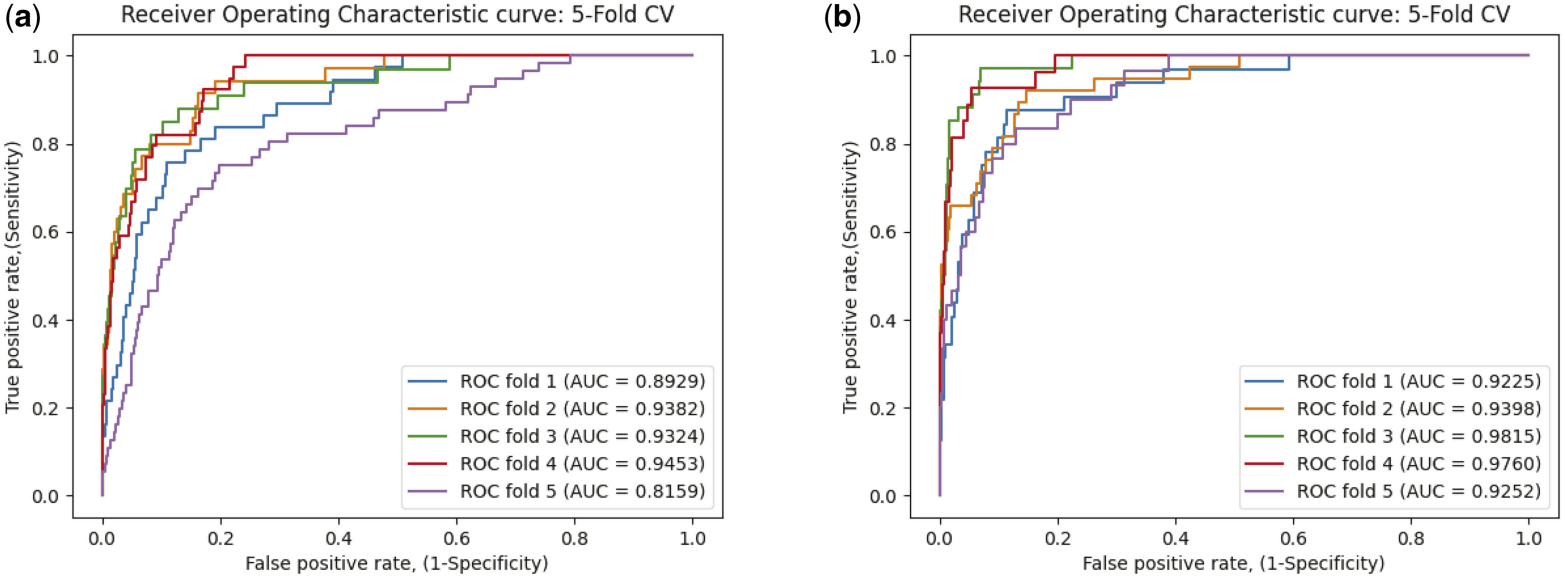
The ROC curves of 5-fold cross-validation by the proposed MDGF-MCEC on the Circ2Disease and CircRNADisease. (a) ROC curve on Circ2Disease; (b) ROC curve on CircRNADisease.

To demonstrate the robustness of MLNGCF, we also evaluate it on Circ2Disease and CircRNADisease databases, the results are given in Table 3. As shown in Fig. 7, ROC curves are drawn for the experimental results on two databases, and their AUCs are calculated. The results on Circ2Disease and CircRNADisease show that MLCNGCF achieves good performance on the two databases, the AUCs are both higher than 0.9. In addition, the metrics reach similar levels to the performance on the CircR2Disease database, which confirms that our method MLNGCF can be applied to benchmark datasets from different data sources.

**Table 3.** Results of 5-fold cross-validation obtained by the proposed MLNGCF on circ2Disease and circRNADisease.

	circ2Disease		circRNADisease	
Validation set	AUC	AUPR	AUC	AUPR
1	89.29	48.95	92.25	51.12
2	93.82	52.30	93.98	52.63
3	93.24	51.78	98.15	67.10
4	94.53	57.27	97.60	65.02
5	81.50	45.79	92.52	53.67
Average	90.48 ± 5.41	51.22 ± 4.26	94.90 ± 2.80	57.91 ± 7.53

### 3.5 Performance comparison of different embedding construction in MLNGCF

Before massage propagation, randomly initialized features are used by Wang et.al as a look-up table in the embedding construction. In MLNGCF, this look-up table is updated by the initial circRNA–disease similarity features.

**Figure 8.**
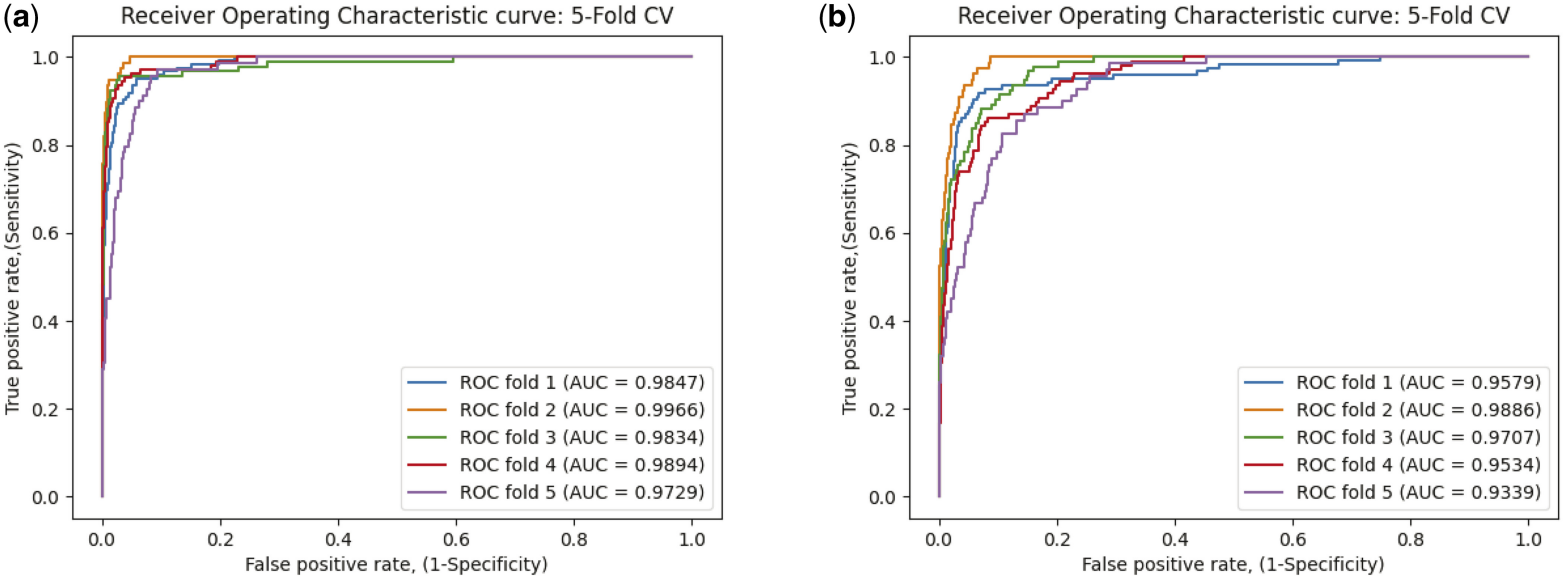
Performance comparison of different embedding construction methods on the circR2Disease database. (a) similarity information as embedding features; (b) randomly initialization as embedding features.

To verify the effectiveness of the look-up table used in MLNGCF, we removed the initial circRNA–disease similarity matrix and replaced it with randomly initialized features as a variant method. The ROC results are shown in Fig. 8, we can see that the AUC of the variant model decreases from 98.54% to 96.06%. The result demonstrates the effectiveness of the similarity matrix as initial lookup tables in MLNGCF.

### 3.6 Effectiveness of multilayer cooperative attention in MLNGCF

**Figure 9.**
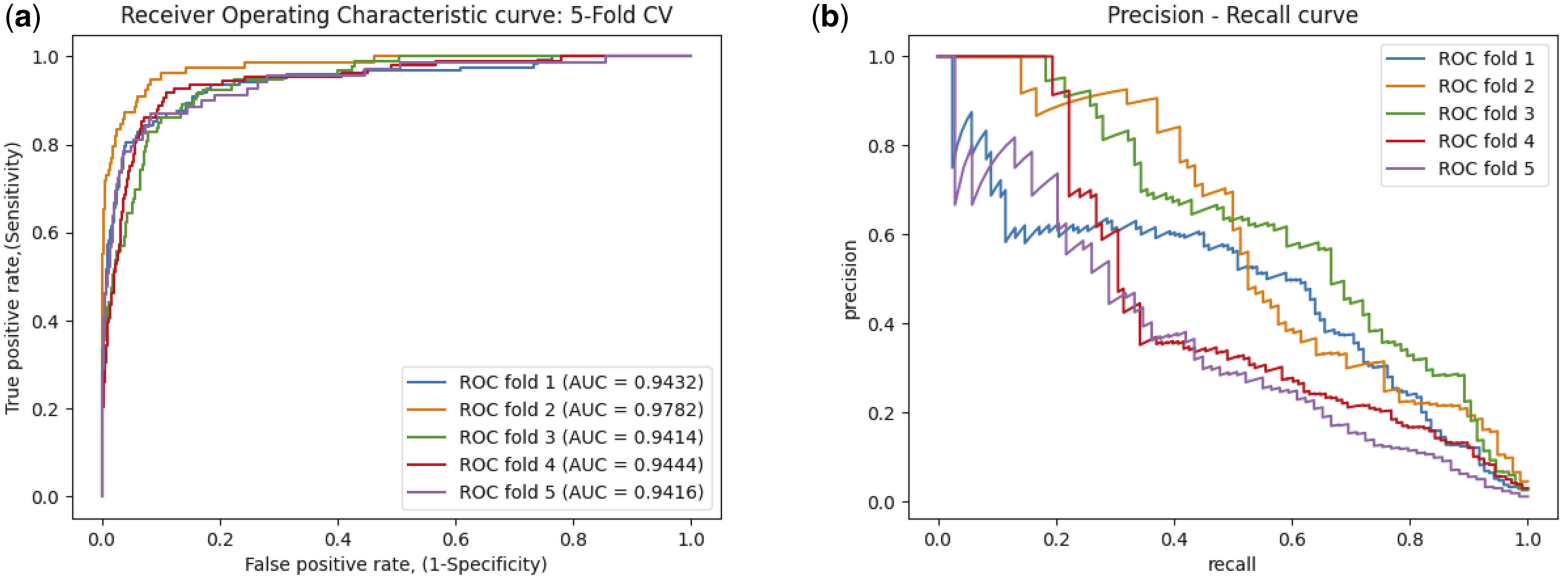
The ROC and PR curves of 5-fold cross-validation on the circR2Disease database without the use of multilayer cooperative attention mechanism. (a) TPR–FPR curve; (b) precision–recall curve.

In order to verify the effectiveness of the multilayer cooperative attention mechanism, we compare MLNGCF with the variant method that removes the multilayer cooperative attention mechanism. The results are shown in Fig. 9. Compared with the original MLCNGCF model (Fig. 5), the AUC and AUPR decreases significantly after removing multilayer cooperative attention heads, where the AUC decreases from 98.54% to 94.87%. The results indicate that multilayer cooperative attention plays an important role in the message propagation process.

### 3.7 Performance comparison of different aggregators in MLNGCF

**Figure 10.**
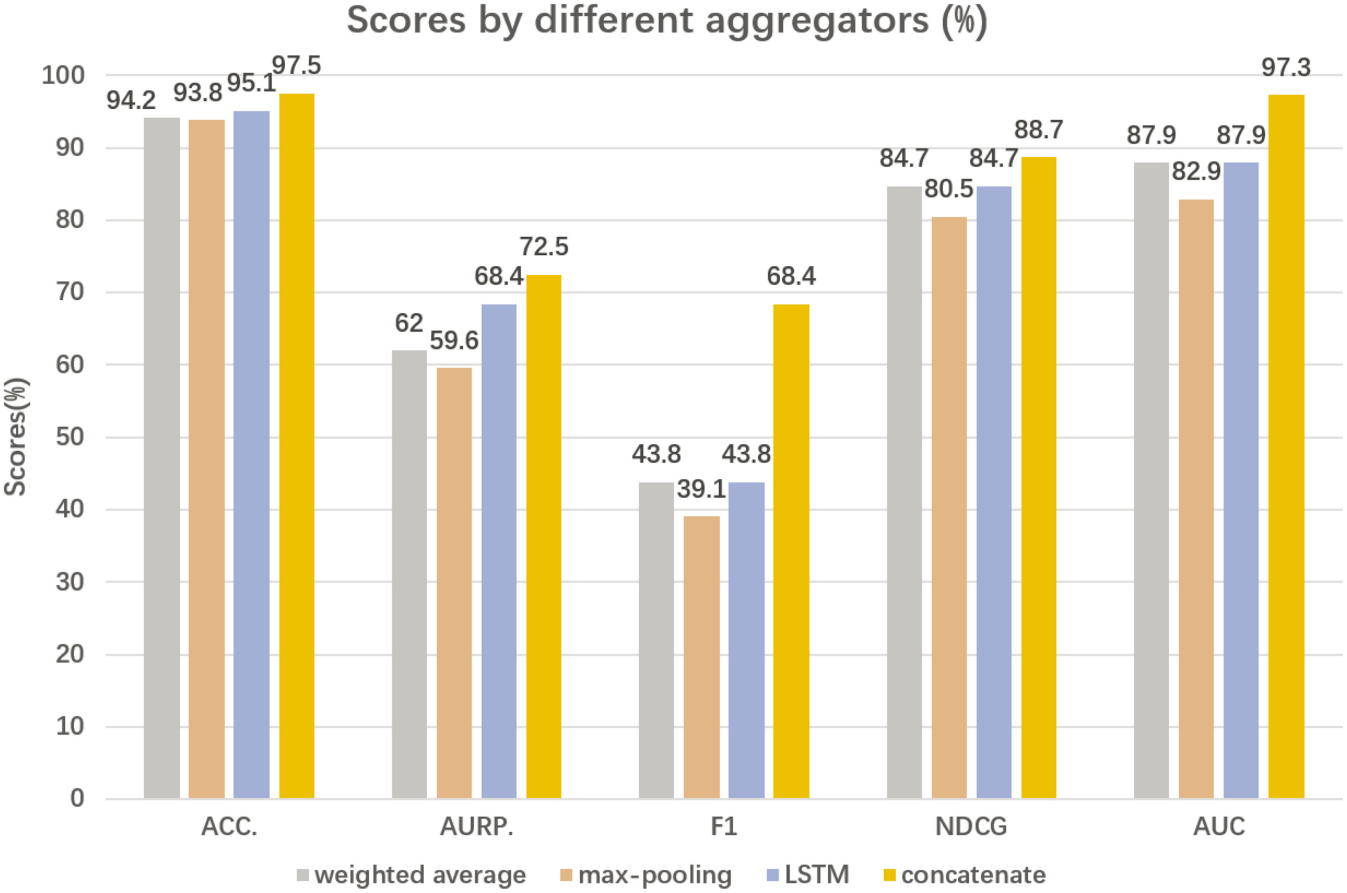
Performance comparison of MLNGCF with different aggregators on circR2Disease database.

In MLNGCF, the embeddings of circRNA and disease obtained from high-order propagation are concatenated for collaborative filtering. Other aggregators such as weighted summation, maximum pooling, LSTM can also be used. In order to verify the most suitable aggregators for MLNGCF, we compare these aggregators in the same configuration and compare the AUCs. The experimental results are shown in Fig. 10. We can see that the MLCNGCF model using concatenation as the aggregator achieves the best AUCs, followed by the maximum pooling, and LSTM.

### 3.8 Number of cooperative attention layers in MLNGCF

For multilayer message propagation, we use three message layers for message propagation and cooperation between multilayer attention heads. Here, we verify that the optimal number of layers is three.

**Figure 11.**
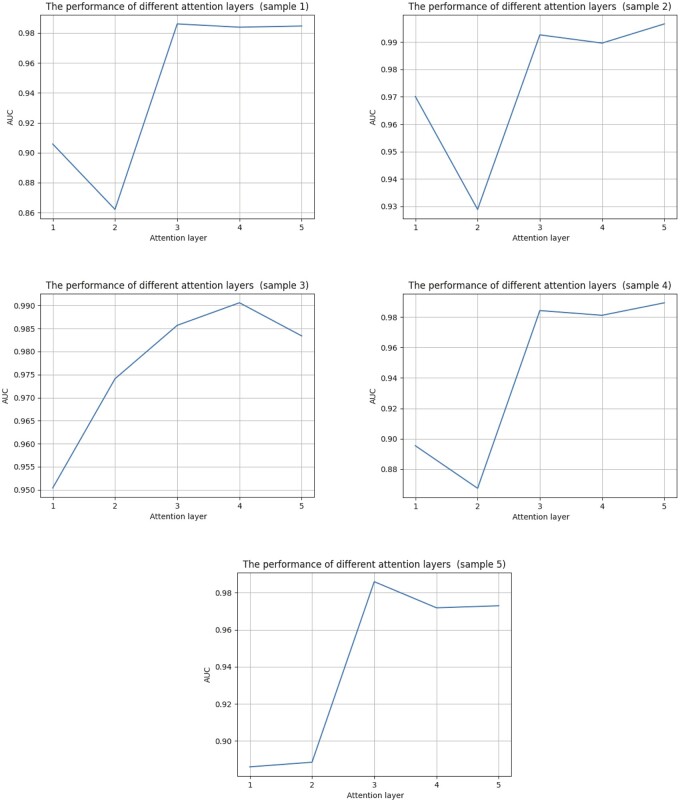
The performance of different attention layers of cooperative attention mechanism on circR2Disease database.

On the CircR2Disease database, the samples are randomly divided into five independent subsets and labeled as Subset 1, Subset 2, Subset 3, Subset 4, and Subset 5 for testing the performance of different numbers of message layers. As shown in Fig. 11, for each sample, the same experimental settings are used from a single message layer to five message layers. As can be seen in the five figures with different message layers, MLNGCF with a single message layer performs the worst, as the number of message layers increases, the model prediction performance shows an increasing trend. We also see that the performance of the model tends to stabilize when the number of message layers is 3. Thus, it can be concluded that MLNGCF with the three message layers and the corresponding multilayer attention mechanism yields the best results.

### 3.9 Case study

To demonstrate the prediction ability of the MLNGCF model, 50,830 unknown associations between 585 circRNAs and 88 diseases are scored by MLNGCF. Here, we search the PubMed database for evidence to support the experimental results. The top 10 circRNAs associated with breast cancer (BC) (Li *et al.* 2019) and hepatocellular carcinoma (HCC) (Fu *et al.* 2018) are listed in Tables 4 and 5. The PMIDs of the support literature are given. For breast cancer, the top candidate hsa_circ_0001946 is experimentally verified by co-expression analysis in early-stage breast cancer tissues (Rao *et al.* 2021).

**Table 4.** The top 10 predicted circRNAs associated with breast cancer.

Rank	Circ-RNA	Disease	Evidence(PMID)
1	CDR1as	BC	31245927
2	hsa_circ_0001649	BC	33544410
3	circRNA_100984	BC	No evidence
4	hsa_circRNA_005019	BC	No evidence
5	hsa_circRNA_102771	BC	No evidence
6	hsa_circRNA_400031	BC	32825956
7	hsa_circ_0005105	BC	No evidence
8	hsa_circ_0084615	BC	No evidence
9	hsa_circRNA_102347	BC	No evidence
10	hsa_circ_0000518	BC	33000910

**Table 5.** The top 10 predicted circRNAs associated with HCC.

Rank	Circ-RNA	Disease	Evidence(PMID)
1	hsa_circ_0024892	HCC	No evidence
2	IQCK	HCC	No evidence
3	hsa_circ_0000519	HCC	36627545
4	EFCAB11	HCC	25665738
5	hsa_circ_0035560	HCC	No evidence
6	hsa_circ_0001400	HCC	30455306
7	GSDMB circRNA	HCC	No evidence
8	hsa_circRNA_002143	HCC	No evidence
9	hsa_circ_0000517	HCC	31750237
10	hsa_circ_0000520	HCC	27258521

The top 10 circRNA–disease pairs are also given in descending order based on prediction scores, and the results are shown in Table 6.

**Table 6.** The top 10 predicted circRNA–disease associations by MLNGCF.

Rank	Circ-RNA	Disease	Evidence(PMID)
1	CDR1as	HCC	33061591
2	hsa_circ_0005567	Cholangiocarcinoma	33062640
3	hsa_circRNA_102619	Alzheimer’s disease	36362022
4	hsa_circ_0061265	OSCC	No evidence
5	hsa_circ_0004846	Endometrial cancer	No evidence
6	hsa_circRNA_103458	Varicosities	29137225
7	hsa_circ_0036722	Atherosclerosis	No evidence
8	circUBAP2	Adenocarcinoma	No evidence
9	hsa_circRNA_005086	Leukoaraiosis	No evidence
10	circRNA-MSR	Cholangiocarcinoma	34581623

## 4 Discussion and conclusion

In this study, we propose *n* novel prediction model MLNGCF, which is a multilayer attention neural graph-based collaborative filtering model for inferring potential circRNA–disease associations. MLNGCF first fuses the similarity information of circRNAs and diseases to construct unified descriptors and refines it by deep auto encoder (DAE). Then, message propagation mechanism is used to exploit key collaborative signals in the adjacency matrix. Attention heads are also constructed at different message layers, and more information can be transmitted during message propagation. In addition, an interaction function of collaborative filtering is introduced to integrate both matrix factorization and MLP and score circRNAs–disease associations. Experimental results verify the effectiveness and reliability of MLNGCF.

However, there are still some limitations that need to be further exploited in the future. On the one hand, the limitation of poorly annotated data affects the generalization ability of the proposed method. On the other hand, the proposed method cannot be applied to new circRNAs and new diseases, because the circRNAs and diseases need be in the network. In the next step, we expect to combine more representative databases and construct more advanced algorithms for similarity calculation, such as novel sequencing techniques and heterogeneous networks of circRNAs and diseases. Considering that MLNGCF treats circRNA–disease pairs with unknown associations as the negative samples, this strategy may result in deviations of the training process. Positive-unlabeled learning which builds a classifier with only positive and unlabeled examples can be introduced.

## Supplementary Material

btad499_Supplementary_Data
