# Supplementary material for: MLNGCF: circRNA–disease associations prediction with multilayer attention neural graph-based collaborative filtering
Source: Bioinformatics. 2023 Aug 10;39(8):btad499. doi: 10.1093/bioinformatics/btad499 (PMC10457666; doi:10.1093/bioinformatics/btad499)
Supplement: btad499_Supplementary_Data [file btad499_supplementary_data.pdf]

Supplementary Material for

MLNGCF: circRNA-disease associations prediction with multi-layer attention neural graph based collaborative filtering

by Wu et al.

Part A: Procedure of disease semantic similarity construction

For example, a disease d can be described as $DAG_d = (d, A_d, E_d)$, where A_d represents the set of all ancestor nodes of d (including d), and E_d is the set of edges among these diseases. If a disease e is in A_d , then its contribution to the disease d can be calculated [1] as

$$\begin{cases} D_d(e) = 1 & if\ e = d \\ D_d(e) = \max\{\varepsilon \cdot D_d(e') | e' \in children\ of\ e\} & if\ e \neq d \end{cases} \tag{1}$$

where ε represents the contribution between the disease e and its child e' and is set as 0.5.

Then, we can calculate the semantic value $DV(d)$ for the disease d as follows:

$$DV(d) = \sum_{e \in A_d} D_d(e) \tag{2}$$

It is assumed that the higher the degree of the overlap among the ancestral diseases shared by two different diseases in DAG, the greater the semantic similarity between the two diseases. Thus, we obtain the disease semantic similarity as:

$$SD_1(d(i), d(j)) = \frac{\sum_{e \in A_{d(i)} \cap A_{d(j)}} \left(D_{d(i)}(e) + D_{d(j)}(e)\right)}{DV(d(i)) + DV(d(j))} \tag{3}$$

where $d(i), d(j)$ represent the *i*-th disease and the *j*-th disease, respectively.

Since the above semantic similarity is focused on the associations between the target disease and its ancestral diseases, it is dominated by the frequency of the target disease in DAG. Another semantic similarity is thus introduced to increase the contribution of rare diseases. The contribution $D'_d(e)$ of a rare disease e to a disease d is defined as follows:

$$D'_d(e) = -\,log\left(\frac{num(DAGs(e))}{num(diseases)}\right) \tag{4}$$

where $DAGs(e)$ represents all the connected subgraphs related to the disease e in DAG, $num(DAGs(e))$ is the number of occurrences of e in DAG, and $num(diseases)$ is the number of diseases in all DAGs.

Therefore, the semantic similarity of the disease $d(i)$ and $d(j)$ is expressed as:

$$SD_2(d(i), d(j)) = \frac{\sum_{e \in A_{d(i)} \cap A_{d(j)}} \left(D'_{d(i)}(e) + D'_{d(j)}(e)\right)}{DV(d(i)) + DV(d(j))} \tag{5}$$

where $DV(d(i))$ denotes the semantic similarity of the disease $d(i)$.

Finally, the two semantic similarities are fused to obtain the disease semantic similarity $D^s(d(i), d(j))$:

$$SD(d(i), d(j)) = \frac{SD_1(d(i), d(j)) + SD_2(d(i), d(j))}{2} \tag{6}$$

Part B: Procedure of disease GIP kernel similarity construction

In order to make the similarity information of disease more comprehensive, Gaussian interaction profile kernel similarity (GIP)[2] is also introduced to measure the similarity between diseases. Based on the hypothesis that similar diseases may be associated with circRNAs with similar functions, the Gaussian interaction profile kernel similarity is used to define the similarity between two diseases as:

$$DGS\left(d(i), d(j)\right) = exp\left(-\lambda \left\| V\left(d(i)\right) - V\left(d(j)\right) \right\|^2\right) \tag{7}$$

$$\lambda = \frac{1}{\frac{1}{N_d}\sum_{i=1}^{N_d} \left\| V\left(d(i)\right) \right\|} \tag{8}$$

where V is the adjacency matrix of circRNAs and diseases based on the CircR2Disease database; $V(d)$ is the row vector in V, whose elements are either 0 or 1, representing the association between disease d and circRNAs; λ is the width parameter of the Gaussian function, and N_d is the number of diseases.

Part C: Procedure of circRNA functional similarity construction

The construction of the functional similarity[3] between two circRNAs is based on the assumption that circRNAs associated with the same disease may have similar functional semantics. Thus, the functional similarity between two circRNAs can be calculated as follows:

$$FC\big(c(i),c(j)\big)=\frac{\sum_{1\le q\le |D(i)|}S\big(d(q),D(j)\big)+\sum_{1\le r\le |D(j)|}S\big(d(r),D(i)\big)}{|D(i)|+|D(j)|} \tag{9}$$

$$S\big(d(q),D(j)\big)=\max_{1\le s\le |D(j)|}\Big(SD\big(d(q),d(s)\big)\Big) \tag{10}$$

where, $c(i)$ and $c(j)$ are the *i*-th and *j*-th circRNA, $D(i)$ and $D(j)$ are the sets of diseases related to the two circRNAs, respectively, and $SD\big(d(q),d(s)\big)$ is defined in Eq.(6).

Part D: Procedure of circRNA GIP kernel similarity construction

Based on the assumption that circRNAs with similar functions may be associated with similar diseases, GIP kernel similarity can represent the similarity between circRNAs as:

$$CGS(c(i), c(j)) = exp\left(-\lambda \left\| V(c(i)) - V(c(j)) \right\|^2\right) \tag{11}$$

$$\lambda = \frac{1}{\frac{1}{N_c}\sum_{i=1}^{N_c}\left\| V(c(i)) \right\|} \tag{12}$$

where V is the adjacency matrix of circRNAs and diseases, $V(c)$ is a column vector in V which represents the corresponding associations between circRNA c and the diseases, λ is a regularization parameter, and N_c is the number of circRNAs. The value of each element in $V(c)$ is either 0 or 1. If a circRNA is related to a certain disease, the corresponding element in $V(c)$ is set to 1; otherwise 0.

Part F: Details of evaluation metrics

The metrics used in this study are defined as follows:

$$Accuracy = \frac{TP+TN}{TP+TN+FP+FN} \tag{13}$$

$$Precision = \frac{TP}{TP+FP} \tag{14}$$

$$recall = \frac{TP}{TP+FN} \tag{15}$$

$$F1 = \frac{2TP}{2TP+FP+FN} \tag{16}$$

where TP, TN, FP and FN are true positives, true negatives, false positives, and false negatives, respectively.

As another metric, NDCG (Normalized Discounted Cumulative Gain) is frequently used in the recommendation system. This metric can also be used to evaluate the gap between the real labels and the predicted labels.

Reference

1. Pesquita C, Faria D, Falcao AO et al. Semantic similarity in biomedical ontologies, PLOS Computational Biology 2009;5:e1000443.
2. Van Laarhoven T, Nabuurs SB, Marchiori E. Gaussian interaction profile kernels for predicting drug–target interaction, Bioinformatics 2011;27:3036-3043.
3. Chen X, Clarence Yan C, Luo C et al. Constructing lncRNA functional similarity network based on lncRNA-disease associations and disease semantic similarity, Scientific reports 2015;5:1-12.
